# The Pre-clinical Toolbox of Pharmacokinetics and Pharmacodynamics: *in vitro* and *ex vivo* Models

**DOI:** 10.3389/fphar.2019.00578

**Published:** 2019-05-24

**Authors:** Carolina Herrera

**Affiliations:** Section of Virology, Division of Infectious Diseases, Department of Medicine, Faculty of Medicine, Imperial College London, London, United Kingdom

**Keywords:** HIV, antiretrovirals, antibodies, pharmacokinetics and pharmacodynamics, pre-clinical models

## Abstract

Prevention strategies against sexual transmission of human immunodeficiency virus (HIV) are essential to curb the rate of new infections. In the absence of a correlate of protection against HIV infection, pre-clinical evaluation is fundamental to facilitate and accelerate prioritization of prevention candidates and their formulations in a rapidly evolving clinical landscape. Characterization of pharmacokinetic (PK) and pharmacodynamic (PD) properties for candidate inhibitors is the main objective of pre-clinical evaluation. *in vitro* and *ex vivo* systems for pharmacological assessment allow experimental flexibility and adaptability at a relatively low cost without raising as significant ethical concerns as *in vivo* models. Applications and limitations of pre-clinical PK/PD models and future alternatives are reviewed in the context of HIV prevention.

## Introduction

The majority of HIV transmissions currently occur via the genital and the colorectal tracts. Following infection of the initial mucosal founder population (Li et al., 2009), viral amplification is essential for irreversible acquisition of infection and takes place in the first few days (1–3 days) after viral exposure, giving a very short window of opportunity to prevent establishment of infection. In the absence of a vaccine and acknowledging that condoms, male circumcision and behavioral interventions appear insufficient to control the epidemic, the development of mucosal prevention strategies remains an important global public health priority. A prevention method should be safe, acceptable, affordable, and capable of inhibiting viral transmission at the mucosal portals of entry. Effectiveness and adherence of prevention candidates can only be evaluated in clinical trials (Heise et al., 2011); however, phase III clinical trials are expensive, time consuming and require a large number of participants to determine efficacy (Douville et al., 2006; Nuttall et al., 2007). Furthermore, with the introduction of FDA-approved prevention interventions, such as Truvada for oral pre-exposure prophylaxis (PrEP) [Centers for Disease Control and Prevention (CDC), 2012], incidence of infection within communities will decrease, closing the window to perform placebo-controlled trials and causing the trials to become even larger to test later-generation products. Hence, pre-clinical models are increasingly important tools to reduce the risk of late stage failure in clinical trials.

A single model capable of providing all the information to prioritize the best-in-class candidate is not currently available. Furthermore, the drug profile required to prevent mucosal transmission has not been defined, nor has the best pharmacologic measure of efficacy been defined for different dosing routes of candidate inhibitor. Hence, the range of *in vivo, ex vivo*, and *in vitro* assays is continuously being expanded to assess parameters such as mechanism of action, potency and selectivity, PK/PD, safety, immune response elicited, stability, formulation, dosing and potential of acceptability, which will facilitate scaling when defining dosing regimens to be tested in humans. Dose-efficacy discrepancies between animal studies and clinical trials have been described (Romano et al., 2013) highlighting the gap in knowledge regarding the concentration-effect relationship in different species and mucosal compartments. This further emphasizes the need to develop models that will allow PK/PD evaluation of candidate inhibitors in the mucosal environment, recapitulate the factors potentially affecting a direct correlation between PK and PD parameters and facilitate appropriate comparisons between animal studies and humans, increasing the predictive capacity of pre-clinical studies.

HIV-inhibitor candidates include antiretrovirals (ARVs) for PrEP and post-exposure prophylaxis (PEP), broadly neutralizing antibodies (bNAbs) delivered by passive immunization (Morris and Mkhize, 2017) and enhancers of the innate mucosal barrier functions (Herrera and Shattock, 2014; Lajoie et al., 2017). The majority of ARVs currently considered for prophylaxis are already used in highly active ARV treatment (HAART); hence, a substantial amount of pharmacological data has been accumulated for these compounds in biological fluids, such as blood plasma and genital secretions (Cohen et al., 2007; Dickinson et al., 2010). However, drug concentration measurements in blood plasma are not representative of mucosal concentrations (Lederman et al., 2004; Cohen et al., 2007; Dumond et al., 2007, 2009; Brown et al., 2011; Trezza and Kashuba, 2014) and mucosal tissues are histologically and immunologically different from blood (Anton et al., 2000), affecting the expected correlation between concentration and efficacy at mucosal sites. In addition, drug accumulation is specific to each mucosal compartment, with differences between the intestinal and the female and male genital tracts (Cohen et al., 2007; Patterson et al., 2011; Louissaint et al., 2013), partially due to tissue-specific expression of drug transporters (Nicol et al., 2014). This review will discuss *in vitro* and *ex vivo* PK and PD models available and in development, their capacity to mimic fundamental aspects of the mucosal environment, their value for different candidates and dosing routes, their limitations and their potential in predicting the outcome of clinical trials (Table 1).

**Table 1:** Applications of current *in vitro* and *ex vivo* models for development of HIV-prevention strategies.

**Model**	**Applications**	**Stage in development pipeline**	**Advantages**	**Disadvantages**
Cell lines	•PD (*in vitro* or *in vivo* dosing) •PK (drug cellular transport) •Safety (cellular toxicity)	Initial screening	•Short experimental procedure (2–7 days) •Sensitive assays •Low cost •Accessible to most laboratories •Easy-to-use protocol •High throughput •Effect of biological fluids can be assessed	•Low physiological relevance •Over-expression of CD4 and/or HIV co-receptors •Some cell lines are not budding competent (inhibitors of viral maturation cannot be tested) •Do not replicate donor-to-donor variation •Cancerous origin
Primary cells	•PD (*ex vivo* or *in vivo* dosing) •PK (drug cellular transport) •Safety (cellular toxicity, cellular responses)	After initial screening in cell lines	•Increased physiological relevance •Express realistic levels of CD4 and HIV co-receptors •Secrete cytokines •Accessible to most laboratories •Mimic donor-to-donor variability •Effect of biological fluids can be assessed	•Longer procedure (minimum 11 days) •Low mucosal relevance •Ethics approval required and, in some cases, signed informed consent from donor •Medium to high protocol complexity •High cost in some cases
Cellular co-cultures	•PD (*ex vivo* or *in vivo* dosing) •PK (drug cellular transport) •Safety (cellular toxicity, cellular responses)	Following initial screening in cell lines and primary cells	•Mimic *trans* infection •Effect of biological fluids can be assessed	•Longer procedure (minimum 11 days) •Low throughput •Ethics approval required •Medium to high protocol complexity
Tissue explants	•PD (*ex vivo* or *in vivo* dosing) •PK (drug cellular transport) •Safety (toxicity and innate responses)	Before *in vivo* safety and/or efficacy studies	•*in vivo*-like complexity and structure •Mimic *in vivo* viral replication fitness •Innate responses can be measured •Migratory can be isolated in some models •Mimic donor-to-donor variability •Mucosal inflammation can be induced •Good correlation with *in vivo* studies •Effect of biological fluids can be assessed •Versatile model •Good data reproducibility through protocol standardization	•Longer procedure (minimum 11 days) •Progressive decay of structure with culture •Ethics approval and signed informed consent from donor required •Fresh tissue preferable •Cannot replace *in vivo* models yet •Limited specimen size •Low throughput •Medium to high protocol complexity

## Pharmacological Parameters for HIV Prevention Strategies

PK describes the time course of drug concentration which is affected by absorption, distribution, metabolism and elimination and can be summarized as what the body does to the drug. PD describes the resulting effect of a drug, its intensity, time course and potential toxicity or responses to the drug, i.e., what the drug does to the body. Pharmacological assays aim to define PK and PD measures which include parameters such as Cmin (minimum concentration achieved within a dosing interval); Cmax (maximum concentration achieved within a dosing interval); C*t* (concentration at a certain time point post-dosing); AUC (area under the curve for drug concentration during a period of time); Tmax (time to achieve maximum concentration); *t*_1/2_ (half-life; time required for concentration to decrease 50%); *k*_*e*l_ (elimination rate over time); MIC (minimal inhibitory concentration); MEC (minimal effective concentration); *T* (time the concentration remains over the MIC or MEC); IC_50_ (50% inhibitory concentration); EC_50_ (50% effective concentration), extent of viral replication at the last time point of the assay or during a period of time (AUC of viral replication readout between two time points); cytotoxicity and immunological toxicity. The two main read-outs required to calculate these parameters are drug concentration and level of infection after treatment of the model with candidate inhibitors. Drug concentration can be measured as cell-free drug (in culture supernatants, in secretions or in plasma) or intracellularly (in cells or tissues) and new analytical methods are constantly being developed to measure the concentration of candidate inhibitors in these different matrixes. Intracellular measurements are necessary for inhibitors that require metabolization for activation such as some reverse transcriptase inhibitors. Evaluation of viral replication is specific for each pre-clinical model and can be done through measurement of a reporter signal, of gag protein (p24 for HIV and p27 for SIV) by ELISA, or of viral RNA/DNA by PCR or qRT-PCR (Berry et al., 2011).

Assays that provide data to calculate PK/PD will be defined by multiple factors including, among others, the candidate inhibitor, the dosing route, the formulation and the anatomical site of action. ARVs and modulators of mucosal immunity can be formulated for oral or topical dosing, as injectables or as implants; bNAbs can be delivered topically, intravenously or intramuscularly for passive immunization. For mucosal prevention, independently of the dosing route, concentrations measured in the genital and colorectal tracts will need to be sufficient to inhibit viral infection, and these concentrations will be tissue-specific.

## Cellular Models

Inhibitory potency of candidate HIV inhibitors is initially screened in *in vitro* models such as cell lines susceptible to HIV infection that allow calculation of PK parameters and evaluation of cellular toxicity. Compounds or Abs are then tested in *ex vivo* cellular models such as PBMCs, which in addition to PK/PD parameters can provide toxicity and immunological safety information.

### Cell Lines

Multiple cell lines are routinely used to screen potential efficacy of compounds and Abs including CD4^+^T cell lines and non-lymphocytic cells that are transfected to express CD4 and CCR5 and/or CXCR4. Among the CD4^−^ parental cell lines there are human glioblastoma cells, U87MG, which stably express human CD4 and CCR5 (U87 CD4^+^ CCR5^+^ cells) or CXCR4 (U87 CD4^+^ CXCR4^+^ cells) (Bjorndal et al., 1997); indicator cells derived from human osteosarcoma cells, HOS, stably transfected with human CD4, CCR5 and/or CXCR4 [GHOST (3) CCR5^+^, GHOST (3) CXCR4^+^ and GHOST (3) CXCR4^+^ CCR5^+^ cells] and that express green fluorescent protein (GFP) upon production of the viral trans-activator of transcription (Tat) (Morner et al., 1999); and human cervical epithelial carcinoma reporter cells, TZM-bl, which are HeLa cells expressing CD4, CCR5, CXCR4 and under control Tat, luciferase and β-galactosidase (Platt et al., 1998, 2009; Derdeyn et al., 2000; Wei et al., 2002; Takeuchi et al., 2008). This later cell line is susceptible to HIV-1, HIV-2, simian immunodeficiency virus (SIV), and simian human immunodeficiency virus (SHIV) and is nowadays one of the main cell lines used to screen inhibitory activity of ARVs and neutralization potency of Abs. TZM-bl cells are a single viral cycle assay model that requires 2 days of culture before infectivity is assessed by measurement of luciferase expression in cell lysates as relative light units or by measurement of absorbance with a β-galactosidase colorimetric assay. However, this cellular model does not allow efficient viral budding (Carlson et al., 2008) and therefore the activity of compounds that block viral maturation such as protease inhibitors cannot be evaluated with this assay (Stefanidou et al., 2012). Furthermore, TZM-bl cells are HeLa cells that endogenously express CXCR4 but express artificially high levels of CD4 and CCR5 (Polonis et al., 2008). Another reporter HeLa cell line, Affinofile (Johnston et al., 2009), resolves this issue by expressing variable levels of CD4 and CCR5 or CXCR4 based on the amount of selection antibiotic used in culture.

Drug screening is often completed in this model with evaluation of inhibitory potency in the presence of relevant mucosal fluids. TZM-bl cells have been also used to evaluate anti-viral activity in trials by incubating these cells with mucosal secretions (Keller et al., 2011; Herold et al., 2016) obtained from PrEP trial participants, or serum and plasma (Montefiori et al., 2012) during vaccine trials. However, biological fluids can decrease or enhance the level of infection measured in this model (Ghosh et al., 2010b; Hughes et al., 2016) due to inhibitory, toxic or enhancing factors in the fluid matrix such as innate molecules, secreted metabolites or chemical compounds taken by the donor. Therefore, dilution and/or filtration of the sample is required to avoid cytotoxic effects and contamination of the culture (Fletcher et al., 2009; Ghosh et al., 2010a; Harman et al., 2012; Mukura et al., 2012; Romas et al., 2014; Jais et al., 2016). No effect has been observed on the susceptibility to infection of TZM-bl cells by the presence of endotoxins in the biological specimens nor with samples obtained at different stages of the menstrual cycle or during pregnancy (Geonnotti et al., 2010; Patel et al., 2014; Hughes et al., 2016); nevertheless, protocols have been developed to avoid artifacts. The innate anti-HIV activity of cationic factors present in cervical secretions can be prevented by selective depletion of cations (Venkataraman et al., 2005). When measuring Ab neutralization potency in serum or plasma, the presence of other HIV-inhibitory factors can be determined by pre-screening the activity of biological specimens in TZM-bl cells against a chimeric HIV-1 virus containing the Env of murine leukemia virus, which will not be recognized by anti-HIV Abs (Sarzotti-Kelsoe et al., 2014a). TZM-bl cells have also been further transfected to develop a model for evaluation of HIV innate responses (Trotard et al., 2016).

Numerous human CD4^+^T cell lines have been used to determine inhibitory potency parameters. Among them, initial models MT-2 (Harada et al., 1985; Haertle et al., 1988) and MT-4 cells (Harada et al., 1985; Pauwels et al., 1987; Larder et al., 1989) expressing HTLV-1 have been progressively replaced by other T cell lines such as CEM-CCRF cells (Foley et al., 1965), PM-1 cells (Lusso et al., 1995) and C8166 cells (Salahuddin et al., 1983; Lee et al., 1984). CD4^+^CXCR4^+^ A3R5 cells have been transfected with CCR5 as a sensitive T cell model for evaluation of neutralization potency of Abs using luciferase reporter HIV-1 infectious molecular clones (Kim et al., 2003; Montefiori et al., 2012; Sarzotti-Kelsoe et al., 2014b). Assays in CD4^+^T cells require at least 7 days of culture, allowing multiple rounds of viral replication, and infectivity is determined by measurement of p24 antigen content in culture supernatants with enzyme-linked immunosorbent assay (ELISA) or luciferase expression when using reporter viral plasmids. CD4^+^T cell lines have also been used to study the mechanism of drug cellular transport and the implications of the PK profile of drug candidates for HIV prevention (Taneva et al., 2015).

Safety of compounds is initially determined in cellular models by assessing the level of potential cytotoxicity via measurement of tetrazolium salt (MTT) cleavage into a blue-colored product (formazan) in viable cells (Slater et al., 1963) or by similar assays of cellular viability. Despite the lack of productive infection, epithelial cell lines represent an important model to study potential toxicity or disruption of epithelium integrity induced by the drug (Dezzutti et al., 2004). To assess the impact of candidates on epithelial permeability, epithelial cells can be cultured on the apical chamber of trans-well systems to measure tight junctions. Drug transporters on mucosal epithelium allow penetration of ARVs in the epithelium to access the submucosal stroma where the initial foci of infection is located (Hu et al., 2015). In colon epithelium drug efflux is mainly mediated by P-glycoprotein (Pgp), multi-drug resistance-associated protein (MRP) and breast cancer resistance protein (BCRP) transporters; and drug uptake is mediated by organic anion transporter OATP2B1 and organic cation transporter OCT1 (Englund et al., 2006; Kis et al., 2010; Drozdzik et al., 2014; Nicol et al., 2014; Mukhopadhya et al., 2016a). In female genital tract expression of efflux [ATP-binding cassette (ABC), BCRP, MRP, and P-gp] and influx [equilibrative nucleoside transporter (ENT), soluble carrier (SLC) and OCT] transporters has been described (Gunawardana et al., 2013; Zhou et al., 2013, 2014; Grammen et al., 2014; Nicol et al., 2014; Hijazi et al., 2015). Hence, this model is also relevant for PK studies to determine the impact of drug transporters in epithelial cells on drug or Ab concentrations when crossing the mucosal epithelial barrier (Konig et al., 2010; Kis et al., 2013; Hoque et al., 2015; Taneva et al., 2015; Swedrowska et al., 2017), to evaluate the potential effect of candidate inhibitors on drug transporters (Reznicek et al., 2017) and to study the safety and efficacy of formulations designed to deliver compounds across the epithelium to the HIV target cells (Kapitza et al., 2007; Zidan et al., 2013). Harvested supernatants from trans-well systems can be used to measure drug concentrations and for PD assays with CD4^+^ cells. Microscopy has been considered to evaluate absorption/excretion and intracellular distribution of formulated drug candidates (Mandal et al., 2015; Costanzo et al., 2016; Holmstock et al., 2018). Available epithelial cell lines include urogenital epithelial cells [e.g., ME-180 (Sykes et al., 1970), HT-3 (Fogh et al., 1977) and HEC-1-A (Kuramoto, 1972)] and colorectal epithelial cell lines [such as Caco-2 (Fogh et al., 1977) and SW837 (Leibovitz et al., 1976)].

The main drawback of cell lines is their homogeneity, which fails to reproduce the cellular diversity of mucosal tissues and to replicate donor-to-donor variability. Another limitation is that the majority have cancerous cell origins and therefore, do not recapitulate a healthy mucosal environment. However, models such as the TZM-bl assay provide a sensitive and cost-effective tool for quickly assessing activity of candidate inhibitors.

### Primary Cells

Primary cells such as lectin-activated peripheral blood mononuclear cells (PBMCs) as well as cells derived from PBMCs, including monocyte-derived macrophages and immature dendritic cells (iDCs), provide more physiologically relevant cellular models for anti-viral activity measurements. These *ex vivo* models involve longer experiments (7 to 14 days) allowing multiple viral replication cycles and therefore, tend to be used after initial assessment in immortalized cell lines. Inhibitory activity, concentration and safety of drugs and Abs have been measured in activated PBMCs following *ex vivo* dosing or in cells obtained from animal studies (Garcia-Lerma et al., 2011; Dobard et al., 2012; Massud et al., 2013; Anderson et al., 2014) and trial participants.

PBMCs provide information about the systemic compartment but are not fully representative of mononuclear cells found in mucosal tissues. Indeed, differences in PK parameters such as Cmax and half-life have been observed between PBMCs and mucosal mononuclear cells isolated from digested mucosal tissues obtained after *in vivo* dosing of NHPs (Garcia-Lerma et al., 2011; Dobard et al., 2012; Massud et al., 2013) and humans (Yang et al., 2014; McGowan et al., 2015). Differences in expression of drug transporter have also been described between circulating and mucosal CD4^+^T cells (Kis et al., 2010; Mukhopadhya et al., 2016b). Another limitation is that PBMCs exhibit anti-HIV-1 activity in the presence of bacterial lipopolysaccharide (LPS), which will therefore, artefactually enhance the inhibitory activity of biological specimens if they contain endotoxins (Geonnotti et al., 2010). However, activated PBMCs express more physiologically relevant levels of CD4 and HIV-coreceptors than transfected cell lines such as TZM-bl cells (Polonis et al., 2008), and secrete cytokines upon infection as mucosal tissues. This model is a more stringent tool for PD evaluation than cell lines with values of anti-viral activity closer to those observed in mucosal tissues than those obtained with *in vitro* cellular models. Hence, PBMCs represent an additional filter in the pre-clinical pipeline of modest cost and are accessible to most laboratories.

Primary epithelial cells can also be isolated (Greenhead et al., 2000) and cultured in trans-well systems as described above for epithelial cell lines, to assess safety, concentration and activity of candidate inhibitor after *ex vivo* dosing (Shen et al., 2018, 2019) and safety of excipients used for mucosal dosing (Hu et al., 2016). These cells can be purchased or isolated from primary tissue which involves access to surgical specimens. Furthermore, these cells are difficult to culture and require very specific protocols. These limitations make this model costly and not as accessible as epithelial cell lines.

### Cellular Co-cultures

Another important model is based on co-cultures of different cell types. This model can be set up with cell lines and/or primary cells directly in contact to replicate the interaction between DCs and CD4^+^T cells during the viral amplification of the “founder population” and subsequent viral dissemination to draining lymph nodes. These co-cultures have been used to measure drug and Ab activity against *trans* infection between primary mature DCs (mDCs) and TZM-bl cells or autologous CD4^+^ T cells (Sagar et al., 2012) and between primary iDCs and PM-1 cells (Hu et al., 2004; Herrera et al., 2016).

Co-cultures of epithelial cells with target cells in a dual-chamber model mimicking trans-epithelial migration of drugs, Abs and virus have been successfully used to assess safety and allow studies of HIV transmission and efficacy of candidate inhibitors. Epithelial cell lines have been co-cultured with target CD4^+^ cell lines such as TZM-bl cells (Pasetto et al., 2014); or with primary PBMCs (Dezzutti et al., 2004; Guedon et al., 2015) or DC (Van Herrewege et al., 2007). Shen et al. have recently shown with co-cultures of primary epithelial cells and fibroblasts from the female genital tract in trans-well systems that the epithelial barrier can accumulate reverse transcriptase inhibitors, tenofovir and tenofovir alafenamide (TAF), and release them to susceptible CD4^+^ cells for several days after dosing (Shen et al., 2018).

## Tissue Explants

The next phase in PK/PD evaluation often utilizes tissue models such as *ex vivo* culture of mucosal tissue explants (Grivel and Margolis, 2009). Explants are obtained as biopsies or as surgically resected tissue which upon arrival at the laboratory are dissected to remove the muscularis and cut into small pieces. Several models have been developed for penile (Fischetti et al., 2009), cervical, vaginal and colorectal tissues including polarized (Collins et al., 2000; Abner et al., 2005; Cummins et al., 2007) and non-polarized systems (Greenhead et al., 2000; Hu et al., 2004; Fletcher et al., 2006; Grivel et al., 2007). In non-polarized models, explants are submerged for *ex vivo* dosing with candidate inhibitor and then with virus for *ex vivo* challenge. After incubation and depending on the type of tissue, explants are transferred either to new plates in submerged conditions for culture of cervicovaginal and penile tissue, or onto gelatin sponge rafts presoaked in media to help maintain the structure of colorectal explants by culturing them at the air-media interface. Non-polarized culture reduces the protective function of the epithelial barrier by exposing target cells on the edges of the explant directly to the virus, and therefore allows PD evaluation in what could be considered the “worst-case scenario,” however it also represents the “best-case scenario” of drug or Ab availability to prevent infection of the target cell.

In polarized models the tissue epithelium is oriented upwards on the apical chamber of a trans-well system and the edges are sealed using agarose, Matrigel^TM^ or surgical glue. These models have been prioritized for evaluation of formulated inhibitors to evaluate the ability of the formulation to deliver drugs or Abs to the target cells across the epithelial barrier and to assess the safety profile of the formulation toward the epithelium. Polarized systems require larger tissue explants for an increased surface exposure to the candidate inhibitor, for a correct orientation of the epithelium and to avoid incorrect sealing of the explant edges.

The different mucosal portals of HIV entry have histological and immunological specificities such as epithelium type, abundance of activated HIV-target cells, drug transporter profile, Ab isotype expression, and pH, among others (Fischetti et al., 2009; Hladik and Hope, 2009; Shacklett, 2009; Hijazi et al., 2015; Taneva et al., 2015; Cheeseman et al., 2016; Mukhopadhya et al., 2016a). The known lower level of viral replication in the female genital tract compared to the colorectum *in vivo* is replicated in the tissue explant model with lower read out values of infection in cervicovaginal explants after challenge with a normalized viral input titer (Lapenta et al., 1999; Anton et al., 2000; Poles et al., 2001; Fox et al., 2016). Furthermore, this model recapitulates (Saba et al., 2013) changes in susceptibility to HIV infection in the female genital tract during the menstrual cycle (Rodriguez-Garcia et al., 2013; Thurman et al., 2016; Boily-Larouche et al., 2019) and menopause (Thurman et al., 2017). Gender or location of tissue excision have not been reported to affect the susceptibility to infection of colorectal explants nor the activity of candidate inhibitors (Anton et al., 2011); nevertheless, lower levels of infection are observed in small intestine explants compared to large intestine tissue (Elliott et al., 2018). The predominant transmission of R5-tropic isolates compared with X4-viruses during sexual intercourse (Salazar-Gonzalez et al., 2009; Grivel et al., 2011) is also replicated in the explant model (Herrera et al., 2009). These traits will affect PK and PD profile of candidate inhibitors and therefore are important for the design and evaluation of prevention strategies and their formulation (Trezza and Kashuba, 2014). In fact, tissue drug levels are not only dosing route-dependent but will be distinct for each tissue and distinct from the systemic compartment (Lederman et al., 2004; Cohen et al., 2007; Trezza and Kashuba, 2014), thus affecting the expected correlation between concentration and efficacy at mucosal sites. This can be reflected with the explant model. Greater concentrations of rilpivirine, a reverse transcriptase inhibitor, are required to inhibit infection in ecto-cervical explants than in colorectal tissue (Dezzutti et al., 2016). The activity of maraviroc, a CCR5-binding entry inhibitor, could only be observed in pre-activated ecto-cervical tissue explants; however, activation of colorectal explants was not necessary although limited inhibition was measured in this tissue. These results reflect the heterogeneity in CCR5 conformation and/or expression in the different mucosal tissues, which cannot be assessed in TZM-bl cells, a model expressing high levels of CCR5 (Fletcher et al., 2016; Herrera et al., 2016). Interestingly, the limited activity of maraviroc observed in tissue explants predicted the lack of efficacy of this drug in oral PrEP NHP studies (Massud et al., 2013) and clinical trials (Fox et al., 2016; Gulick et al., 2017; McGowan et al., 2019) despite accumulation of the drug in the mucosal compartments.

Viability of explants has been questioned. However, despite progressive decay in structure, CD4/CD8 cell ratios remain constant and viral replication is sustained (Fletcher et al., 2006). Tissue explants can be kept in culture for more than 3 weeks although for PK/PD evaluation, cultures are kept for 15 days; except for evaluation of protease inhibitors targeting late stages of the viral replication cycle, when explants are cultured for 21 days. During this period, cultures are fed at different time points by harvesting part of the supernatant and adding fresh media. To mimic pulse exposure to drug or Ab, after incubation explants are washed to remove unbound inhibitor and virus and cultured in media without compound; however, to evaluate sustained release systems such as vaginal rings, implants or injectables, after initial incubation and washes, culture media is supplemented with candidate inhibitor during the entire period of culture (Harman et al., 2012; Stefanidou et al., 2012; Fletcher et al., 2016; Zhang et al., 2017).

In cervical and penile tissue models, cells emigrating from the tissue have been described and these can be cultured separately from the explant and in the presence of CD4^+^T cells, such as PM-1 cells, to assess anti-viral activity of a drug against dissemination by migratory cells (Hu et al., 2004; Fischetti et al., 2009).

To further model the PK/PD profile of a candidate inhibitor in a mucosal compartment and during intercourse, mucosal secretions can be added to the tissue explants. Addition of semen or seminal fluid does not affect the activity of reverse transcriptase inhibitors (Neurath et al., 2006; Fletcher et al., 2009; Dezzutti et al., 2012b). Female genital tract secretions can be obtained as cervicovaginal lavages, which dilute the secretion, or as undiluted fluid with Weck-cel spears, vaginal aspirators or with Instead Cups; however, sparse volumes tend to be obtained. In addition, immune factors in secretions will vary during the menstrual cycle (Birse et al., 2015), other pathologies will modulate the level of inflammation (Roberts et al., 2012; Kaul et al., 2015; Introini et al., 2017a,b; McKinnon et al., 2018), microbial content will not be homogenous among women (Pyles et al., 2014; Klatt et al., 2017; Bayigga et al., 2018; Taneva et al., 2018) and hormonal contraception might increase susceptibility to HIV(Morrison et al., 2015) affecting the PK/PD profile. Hence, modeling the viscosity, pH and osmolarity of the female genital tract secretions, synthetic vaginal fluid (Owen and Katz, 1999) and synthetic cervical fluid (Burruano et al., 2002) have been used in tissue assays as pre-clinical alternatives (Fletcher et al., 2009).

Safety of candidate inhibitors and their formulations can also be pre-clinically evaluated in tissue explants. Clinical trials testing the first generation of topical inhibitors revealed the importance of mucosal safety following enhancement of infection (Honey, 2007; Adams and Kashuba, 2012). Cytotoxicity can be easily measured in tissue explants by the MTT viability assay. Immunological safety biomarkers have been defined for mucosal compartments (Fichorova et al., 2004; Fields et al., 2014). The explant model allows evaluation of mucosal responses to candidate inhibitor exposure by measurement of cytokine modulation (Beer et al., 2006; Gali et al., 2010; Zhang et al., 2017). Pre-clinical safety evaluation has allowed optimization of formulations for different mucosal compartments requiring, for example, modification of the osmolarity of a vaginal gel for rectal application (Rohan et al., 2010; Dezzutti et al., 2012a), which was then found to be safe during clinical trial testing (Anton et al., 2012; McGowan et al., 2013).

*Ex vivo* modeling of the mucosal compartment provides efficacy, concentration, and safety data. Additionally, tissue explants recapitulate the viral replication fitness of wild type and resistant isolates observed *in vivo* in patients (Abraha et al., 2009; Herrera et al., 2009) strengthening the predictive potential of this model in the context of increasingly prevalent ARV-resistance (Pennings, 2013; Snedecor et al., 2014). It is estimated that in high-income countries, 10–20% of new infections are caused by ARV-resistant isolates harboring mutations that confer resistance to at least one of the three main types of ARV drugs (Salomon et al., 2000; Briones et al., 2001; Duwe et al., 2001; UK Collaborative Group on Monitoring the Transmission of HIV Drug Resistance, 2001; Little et al., 2002; Chaix et al., 2003; Mendoza et al., 2003; Weinstock et al., 2004).

Studies in NHPs delivered proof of principle that efficacy of topical dosing with tenofovir against rectal challenge could be replicated by *ex vivo* challenge of tissue resections obtained from NHPs topically dosed *in vivo* (Cranage et al., 2008). In fact, this approach of *ex vivo* challenge of mucosal biopsies is increasingly being used as an endpoint of *ex vivo* efficacy of PrEP (Anton et al., 2012; Harman et al., 2012; Richardson-Harman et al., 2012, 2014; McGowan et al., 2015, 2019; Fox et al., 2016) and vaccine trials (Herrera et al., 2014). This model can be used with cervicovaginal samples frozen at the trial sites and thawed at a centralized facility for *ex vivo* challenge (Gupta et al., 2006; Lackman-Smith et al., 2008); however, it requires the use of fresh tissue when assessing efficacy in the colorectal tract (McGowan et al., 2012).

Despite the variety of explant models, it has been shown that consistent results of anti-viral efficacy can be obtained among different laboratories through protocol standardization for a same model (Richardson-Harman et al., 2009).

The tissue explant model will need to be further developed to assess the PK/PD profiles of new inhibitors and their formulations designed to provide long term efficacy. This will require the model to be adapted physically with protocols that will mimic, for example, mucosal efficacy of injectables; and define new biomarkers of safety and activity. New models should also be able to evaluate broad spectrum anti-viral drugs and compounds designed to maintain mucosal health.

## Future Models

The development of engineered human tissues as a model to study physiological functions and pathologies could lead to new systems for safety and PK evaluation of candidate HIV-inhibitors and ideally for efficacy studies. Initial models mimicking the intestinal epithelium were based on the culture of isolated intestinal crypts with human adult stem cells (Sato et al., 2009) embedded in a matrix of Matrigel^TM^ or silk (Chen et al., 2017). Cultures derived from the small intestine are referred to as enteroids and those from colon are known as colonoids; they mimic a three-dimensional functional epithelial barrier capable of eliciting innate immune responses (Chen et al., 2017). Another option is the use of human inducible pluripotent stem cells which differentiate and form spheroid structures that are cultured on a matrix and are known as organoids (Spence et al., 2011; Miura and Suzuki, 2018). However, organoids have fetal and immature phenotypes and therefore, a certain degree of maturity can be obtained during culture as shown with liver organoids (Takebe et al., 2013). To increase the physiological relevance of these models, fluidic devices have been incorporated into models known as “organ-on-a-chip” or “microphysiological systems.” Human gut-on-a-chip systems were developed originally using Caco-2 cells (Kim et al., 2012). This represents a structurally oversimplified model lacking immune cells and not achieving fully mature adult phenotypes. However, the field is constantly evolving and new models combining organoid and organ-on-a-chip technologies provide primary gut chips (Kasendra et al., 2018). The limited structural resemblance of these devices with *in vivo* tissue could be resolved using three-dimensional bioprinting techniques (Mittal et al., 2018). Other drawbacks are the microfluidic and chip costs, the complexity of the microengineering and the cytotoxicity induced by defective flow rates. The greater complexity of the female genital tract compared to the gut cannot be modeled with a unique chip but rather by including multiple organs-on-a-chip in one microphysiological system (Loskill et al., 2015; Edington et al., 2018) that can be used to study the biology and pathogenesis of the female genital tract (Young et al., 2017).

These and future models will need to fully recapitulate the cellular diversity of mucosal tissues, the immune responsiveness and the donor-to-donor variation to provide pre-clinical PK and PD information on candidate HIV inhibitors.

## Conclusions

Pre-clinical assays for HIV prevention remain critical to understanding the relative potential of new compounds and combinations and for selecting the best candidates, their formulation and dosing regimen. Furthermore, in an era where HIV cure research has been prioritized, pre-clinical models developed for prevention might be applicable for the evaluation of cure strategies. However, all pre-clinical assays have their limitations and their value in predicting clinical efficacy has yet to be established. Hence, the process of product prioritization needs to be based on a range of criteria that include: *in vitro* drug potency, animal efficacy data, stage of product development, cost of goods, existing safety data, and ability to measure PK/PD parameters in clinical trials. New PK/PD parameters or correlations might need to be defined to pre-clinically predict the outcome of clinical trials. This will require further development of existing models, which have not significantly changed in the last decade, and introduction of new models in the pre-clinical toolbox. Ultimately, validation of *in vitro* and *ex vivo* models will require *in vivo* studies in humans.

## Author Contributions

The author confirms being the sole contributor of this work and has approved it for publication.

### Conflict of Interest Statement

The author declares that the research was conducted in the absence of any commercial or financial relationships that could be construed as a potential conflict of interest.
